# A Rare Case of Complete Atrioventricular Block Induced by Lithium Toxicity: Impact of Drug Interactions With Telmisartan

**DOI:** 10.7759/cureus.77402

**Published:** 2025-01-13

**Authors:** Chien-Yuan Chen, Bo-Jun Chang, Nien-Mu Chiu

**Affiliations:** 1 Department of Psychiatry, Kaohsiung Chang Gung Memorial Hospital and Chang Gung University College of Medicine, Kaohsiung, TWN

**Keywords:** bipolar disorder treatment, complete atrioventricular block, lithium-antihypertensive interaction, lithium-induced bradycardia, lithium therapy

## Abstract

Lithium is primarily associated with gastrointestinal and neurological side effects, while, to the best of our knowledge, cardiac toxicity is rarely reported. We present a unique case of lithium toxicity manifesting as a complete atrioventricular (AV) block, occurring without typical signs of lithium toxicity such as tremors, gastrointestinal disturbances, or altered mental status. A 55-year-old woman with bipolar disorder and hypertension presented with worsening mood lability and an increasing frequency of panic attacks. Lithium therapy was initiated to stabilize her mood and manage worsening psychiatric symptoms. However, while receiving treatment in the acute ward, she developed bradycardia with complete AV block, despite the absence of neurological symptoms or any reported discomfort. Elevated serum lithium levels strongly suggested lithium toxicity as the underlying cause. As lithium levels decreased, her heart rate normalized, and her sinus rhythm was restored. A review of her medication regimen identified the antihypertensive agent telmisartan as a potential contributor to lithium toxicity, which ultimately resulted in severe arrhythmia. Following her recovery, a detailed evaluation with a 24-hour Holter electrocardiogram (ECG) revealed no significant arrhythmias or pauses, further supporting the conclusion that the bradycardia was primarily due to the interaction between lithium and antihypertensive medications. This case emphasizes the importance of recognizing atypical presentations of lithium toxicity, particularly cardiac manifestations, and highlights the potential for drug interactions with antihypertensive agents. Clinicians should maintain vigilance and ensure close monitoring when co-prescribing these medications to mitigate associated risks.

## Introduction

Lithium is widely used in the treatment of bipolar disorder due to its proven effectiveness in stabilizing mood. However, its narrow therapeutic index necessitates regular monitoring to avoid toxicity. The therapeutic range for lithium is 0.6-1.2 milliequivalents per liter (mEq/L), with toxicity typically occurring at concentrations above 1.5 mEq/L [1]. While gastrointestinal and neurological side effects are well-documented, severe cardiac toxicity, such as complete atrioventricular (AV) block, is rare but potentially life-threatening [2].

Lithium toxicity can be exacerbated by interactions with certain antihypertensive medications, particularly those that alter renal function, such as angiotensin-converting enzyme (ACE) inhibitors and angiotensin receptor blockers (ARBs). These agents can reduce the glomerular filtration rate or enhance tubular reabsorption of lithium, resulting in elevated serum lithium levels [3]. Studies have shown that ARBs may increase the risk of lithium intoxication by as much as sevenfold [4]. These interactions highlight the necessity of comprehensive monitoring during lithium therapy, encompassing frequent serum lithium testing at least weekly [5], periodic renal function evaluations, and cardiac assessments such as ECGs to detect early arrhythmias or conduction abnormalities.

Cardiac manifestations of lithium toxicity span a broad range, from sinus node dysfunction, QT prolongation, and T-wave inversions to more severe arrhythmias such as complete AV block and ventricular tachycardia [2]. These complications are thought to arise from lithium's inhibition of voltage-gated sodium channels in myocardial cells, which leads to myocardial electrical instability [6]. Although rare, the life-threatening nature of these cardiac events underscores the importance of heightened clinical vigilance for lithium-induced cardiotoxicity, facilitating prompt diagnosis and intervention to prevent adverse outcomes [6,7].

This case describes the rare occurrence of complete AV block as a manifestation of lithium toxicity, likely exacerbated by an interaction with telmisartan. Notably, the absence of typical symptoms, such as nausea or conscious disturbance, highlights the importance of recognizing atypical and severe cardiac complications, particularly in patients on multiple medications.

## Case presentation

A 55-year-old married woman, diagnosed with bipolar disorder and borderline personality disorder in 2002, presented to the psychiatric outpatient department for evaluation. Her medical history included hypertension managed with telmisartan 80 mg and amlodipine 5 mg daily for over a year, as well as a severe traffic accident in 2023 that resulted in the amputation of her left foot. Post-accident evaluations ruled out traumatic brain injury, and the patient denied any history of previous head trauma or seizures. Her most recent psychiatric admission was in 2021, after which she reported good overall condition and fair self-care ability upon discharge. In the years following, she remained under regular psychiatric follow-up and was prescribed a regimen of sodium valproate 1500 mg/day, quetiapine 750 mg/day, and estazolam 4 mg/day.

In the outpatient department, the patient reported experiencing irritability, hyperactivity, racing and expansive thoughts, impulsive behaviors, and nearly daily emergency room visits for anxiolytic drug injections to manage intermittent panic attacks over the past two weeks. Given her worsening symptoms and inadequate response to the current regimen, lithium 300 mg TID (900 mg/day) was initiated, and hospitalization was recommended to stabilize her condition and achieve symptom remission.

**Figure 1:**
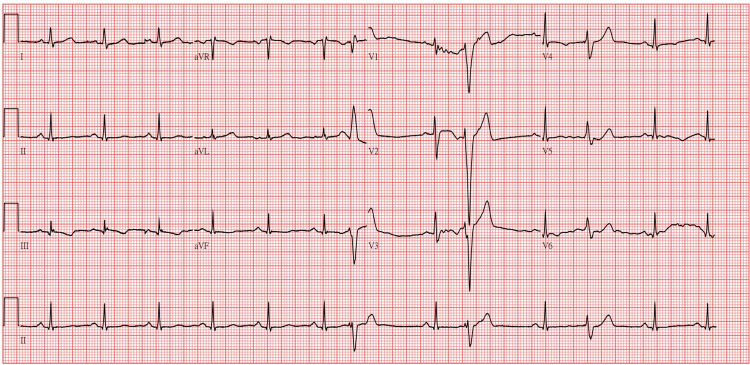
Electrocardiogram on the first day of admission. Initial ECG demonstrates sinus rhythm with frequent premature ventricular complexes. The patient was asymptomatic, reporting no palpitations or chest discomfort.

On the first day of admission, laboratory examinations revealed normal white blood cell count, liver and kidney function, and stable electrolyte levels. Blood pressure was recorded at 137/95 mmHg. The initial ECG showed a heart rate of 76 bpm with sinus rhythm and frequent premature ventricular complexes (Figure 1). Notably, the patient reported no symptoms, including palpitations or chest discomfort. Lithium therapy was initiated with weekly blood monitoring planned to assess its concentration. By day seven, the serum lithium level had risen to 1.23 mEq/L, slightly exceeding the therapeutic range of 0.6-1.2 mEq/L. Physical examination revealed stable vital signs and no signs of lithium toxicity, such as tremors, gastrointestinal disturbances, or altered mental status. The patient reported a gradual improvement in mood stability and a reduction in anxiety symptoms. As a result, the lithium dosage of 300 mg TID (900 mg/day) was maintained.

**Figure 2:**
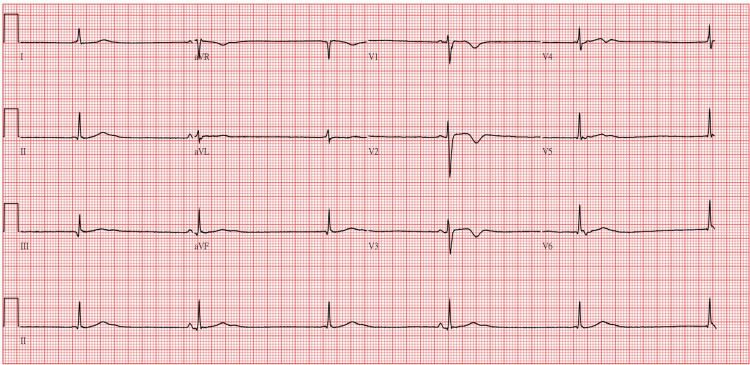
Electrocardiogram during bradycardia episode. ECG performed on the 14th day of admission revealed complete atrioventricular (AV) block, a junctional escape rhythm, and T-wave inversion, correlating with the observed bradycardia and an elevated serum lithium level of 2.47 mEq/L.

On the 14th day of admission, bradycardia with a heart rate of 33 bpm and blood pressure of 103/58 mmHg was observed. However, the patient reported no discomfort and had a Glasgow Coma Scale (GCS) score of E4V5M6, indicating full alertness and orientation. Physical examination showed normal muscle strength (5/5) in the upper and lower extremities and no signs of neurological deficits. A comprehensive cardiac evaluation was initiated to investigate the cause of bradycardia, including an ECG and an assessment for potential electrolyte imbalances or medication interactions contributing to the condition. ECG findings indicated a complete AV block pattern, junctional escape rhythm, and T wave inversion (Figure 2). Laboratory tests revealed a serum lithium level of 2.47 mEq/L with normal electrolyte levels, CBC, thyroid functions, kidney and liver functions, suggesting that the elevated lithium concentration likely contributed to the observed bradycardia and conduction abnormalities.

**Figure 3:**
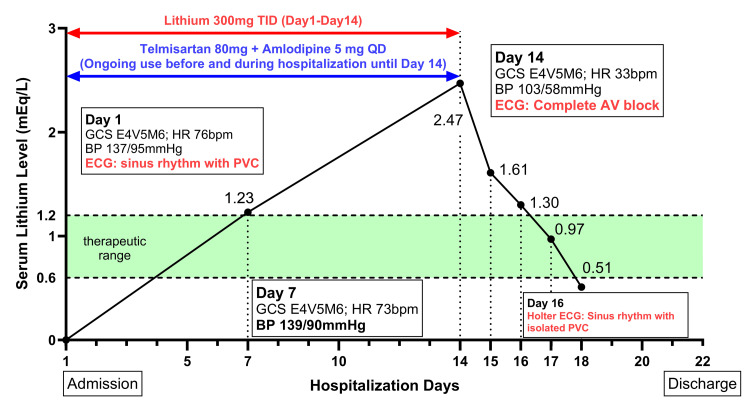
Changes in serum lithium levels and clinical findings during hospitalization. This figure depicts the changes in serum lithium levels (mEq/L) over the course of hospitalization and key clinical findings. Lithium therapy (300 mg TID) was initiated on Day 1 and discontinued on Day 14 due to elevated serum lithium levels (2.47 mEq/L) and the development of bradycardia with complete atrioventricular (AV) block, as confirmed by ECG. Antihypertensive medications (telmisartan 80 mg QD and amlodipine 5 mg QD), which were part of the patient’s long-term blood pressure management regimen, were continued from prior to admission until Day 14. Serum lithium levels decreased following discontinuation of lithium therapy and supportive management, including hydration. By Day 16, Holter ECG revealed sinus rhythm with isolated premature ventricular contractions (PVCs). The green shaded area represents the therapeutic range for serum lithium (0.6–1.2 mEq/L). Clinical findings, including the Glasgow Coma Scale (GCS), heart rate (HR), and blood pressure (BP), are annotated at key time points.

Immediate management included discontinuation of lithium therapy and antihypertensive medications, initiation of adequate hydration with normal saline infusion, and close cardiac monitoring through telemetry. Potential interventions, such as transvenous pacing or atropine administration, were considered if symptoms worsened or hemodynamic instability developed. The patient’s heart rate gradually improved to 55-75 bpm and returned to sinus rhythm as serum lithium levels steadily declined, measuring 1.30 mEq/L, 0.97 mEq/L, and 0.51 mEq/L on consecutive days (Figure 3). Upon further evaluation, it was noted that the antihypertensive agent telmisartan might have contributed to the bradycardia. These medications, particularly in combination with elevated lithium levels, can exacerbate bradycardia by affecting autonomic regulation and further slowing cardiac conduction.

A detailed workup was conducted to evaluate potential causes of bradycardia, including a 24-hour Holter ECG to monitor for arrhythmias, pauses, or conduction abnormalities. The Holter ECG revealed sinus rhythm with isolated premature ventricular contractions (nine beats) and premature atrial contractions (172 beats), both showing minimal overall burdens (<0.1% and 0.3%, respectively). Notably, no atrioventricular conduction block or dynamic ST changes were identified.

These findings suggest that the bradycardia with complete AV block was primarily attributed to the interaction between lithium and telmisartan, resulting in sinus nodal dysfunction rather than intrinsic atrioventricular conduction abnormalities. Additionally, the patient was managed entirely within the inpatient psychiatric unit, where comprehensive cardiac monitoring and treatment were conducted without the need for transfer to another medical floor. The patient was discharged on day 22 with a stable mood and no residual complications from lithium intoxication (Figure 3). The discharge medications included lithium 600 mg/day, sodium valproate 1500 mg/day, quetiapine 800 mg/day, and estazolam 4 mg/day. Notably, antihypertensive medications, including telmisartan, were strictly discontinued due to the potential for drug interactions. The patient was referred to a cardiology clinic for further evaluation and adjustment of antihypertensive therapy.

## Discussion

Lithium has been effectively used for the treatment of mood disorders but can cause severe toxicity when serum levels exceed the therapeutic range [8]. The current case presents a rare but serious complication of lithium therapy, highlighting the importance of recognizing cardiac toxicity as a potential manifestation. While gastrointestinal and neurological symptoms are commonly associated with lithium toxicity, the presentation of complete AV block in this case is unusual, particularly in the absence of classic signs of intoxication. The role of telmisartan in exacerbating lithium toxicity by impairing lithium clearance further complicates the clinical scenario. Although quetiapine and valproate are known to cause hypotension and other cardiovascular adverse effects [9,10], the patient had been on long-term therapy with both medications without any history of such complications. Thus, it is unlikely that the cardiovascular events observed in this case were attributable to either drug. This case underscores the need for heightened vigilance when prescribing lithium in patients with comorbid hypertension requiring antihypertensive therapy.

Lithium toxicity typically manifests at serum levels >1.5 mEq/L with gastrointestinal and CNS symptoms, while levels >2 mEq/L may lead to seizures, coma, or fatal outcomes. Cardiac complications associated with lithium toxicity are generally observed when serum lithium levels exceed 1.5 mEq/L, with severe toxicity typically occurring at levels >2.5 mEq/L [5,11]. Mild cases of lithium toxicity are managed with lithium discontinuation and hydration, whereas severe cases with levels >3 mEq/L or hemodynamic instability may require hemodialysis [12]. Transvenous pacing is reserved for refractory bradyarrhythmias with complete heart block or hemodynamic compromise [1,12]. Additionally, case reports have shown complete AV block occurring at lithium levels as high as 2.2 mmol/L and 3.8 mmol/L [11,13]. In the current case, the patient’s serum lithium level of 2.47 mEq/L and complete AV block were effectively managed with supportive therapy, including lithium discontinuation and hydration, without the need for hemodialysis or pacing.

Previous studies have reported that lithium can cause various ECG changes, including second-degree type II and third-degree heart blocks [2,14]. The proposed mechanism involves a reduction in intracellular potassium due to inhibition of myocyte voltage-gated sodium channels, leading to myocardial electrical instability [15,16]. At therapeutic serum lithium levels, sinus node dysfunction and T-wave inversions are the most commonly observed abnormalities. However, at toxic levels, more severe arrhythmias can develop, including sinus and AV node dysfunction, complete heart block, and even myocardial infarction [2,17]. In this case, the patient presented with asymptomatic bradycardia, with ECG findings showing complete AV block, a junctional escape rhythm, and T-wave inversions. This case emphasizes the potential for abrupt and severe cardiac complications related to lithium toxicity, even in the absence of typical neurological or gastrointestinal symptoms.

The combination of lithium with antihypertensive agents, particularly ACE inhibitors and ARBs, poses significant risks due to their potential to exacerbate lithium toxicity [4]. Studies have shown that ARBs can increase the risk of hospitalization for lithium toxicity by sevenfold [4]. Telmisartan, a widely used ARB, has been implicated in lithium intoxication by reducing lithium clearance in the proximal tubules through its impact on renal function [3,18]. These findings underscore the importance of identifying and managing drug interactions between lithium and antihypertensive agents to prevent atypical and potentially life-threatening complications.

Current guidelines emphasize the importance of frequent and proactive monitoring of serum lithium levels, particularly during the initiation or adjustment of ARB therapy [5]. Lithium is usually started at low doses, such as 400 mg daily, with the dose titrated up to achieve serum lithium levels between 0.5 and 1.2 mmol/L based on the clinical response and adverse effects [5,19]. Specifically, baseline lithium levels should be measured prior to starting or modifying ARB dosages, with follow-up assessments conducted five to seven days after adjustment and continued on a weekly or biweekly basis until levels stabilize. Once stability is achieved, more frequent monitoring intervals (e.g., every three months) are recommended for high-risk patients receiving both medications [5,20]. In this case, a comprehensive monitoring plan was implemented, which included weekly serum lithium evaluations and daily assessments for early signs of toxicity, such as tremors, confusion, or gastrointestinal symptoms.

## Conclusions

This case illustrates the rare and potentially life-threatening occurrence of complete AV block as a manifestation of lithium toxicity, particularly in the absence of classic signs such as gastrointestinal or neurological disturbances. The elevated serum lithium levels, likely exacerbated by reduced renal clearance caused by telmisartan, were identified as the primary contributors to the observed cardiac toxicity. Effective management, including the discontinuation of lithium and antihypertensive therapy, hydration, and close cardiac monitoring, successfully resolved the bradycardia without the need for hemodialysis or pacing.

This case highlights the importance of individualized treatment strategies for patients on lithium therapy, particularly those with comorbid conditions requiring antihypertensive medications. Comprehensive monitoring protocols should encompass weekly serum lithium level assessments, periodic renal function evaluations, and cardiac monitoring, particularly during therapy initiation or dose adjustments. Clinicians should be concerned when prescribing combinations of antihypertensive agents with mood stabilizers and antipsychotics, given the potential for complex drug interactions. Maintaining vigilance for atypical and severe cardiac complications is essential to prevent adverse outcomes. Further research is needed to deepen the understanding of lithium-antihypertensive interactions and to establish clinical guidelines for managing such cases.
